# On the Reproduction and Reunion of Divided Tendons

**Published:** 1870-09

**Authors:** M. Demarquay


### On the Reproduction and Reunion of Divided Tendons.

By M. Demarquay.

From the researches of M. Demarquay, it results that neither the blood, nor the plastic lymph, nor the blastema, which have been in succession invoked as elements of reparation, play the part that has been attributed to them.

From these researches the following conclusions have been derived :—

1. That the tendon is regenerated by the proliferation of the elements which are found on the internal surface of the sheath of the divided tendon, the two ends of which are retracted.

2. That the external portion of the sheath remains perfectly indifferent during this phenomenon, except it be that the vessels which it supports become more voluminous and increase in number.

3. That the proliferation which takes place on the internal surface of the sheath takes place at the expense of the cellular elements of this part, which at the end of the eighth or tenth day become confounded with the cellular elements springing from the extremity of the divided tendon.

4. That the regeneration of the tendon is so much the more rapid as the sheath of the divided tendon is more vascular; in fact, while the tendo Achilis is repaired by the twentieth or twenty-fifth day, the ligamentum patellæ requires a more considerable time.

5. That the phenomenon which constitutes the reproduction of tendon is, in all points, conformable to that which takes place in the reproduction of bone from periosteum, a phenomenon which has been well studied by MM. Flourens, Ollier and Sèdillot.—*Half Yearly Abstract.*

